# Population genetic isolation and limited connectivity in the purple finch (*Haemorhous purpureus*)

**DOI:** 10.1002/ece3.2524

**Published:** 2016-10-21

**Authors:** Colin Bran Alexander Macfarlane, Libby Natola, Mike W. Brown, Theresa M. Burg

**Affiliations:** ^1^ University of LethbridgeLethbridgeABCanada

**Keywords:** barriers, phylogeography, Pleistocene, population isolation, refugia

## Abstract

Using a combination of mitochondrial and z‐linked sequences, microsatellite data, and spatio‐geographic modeling, we examined historical and contemporary factors influencing the population genetic structure of the purple finch (*Haemorhous purpureus*). Mitochondrial DNA data show the presence of two distinct groups corresponding to the two subspecies, *H. p. purpureus* and *H. p. californicus*. The two subspecies likely survived in separate refugia during the last glacial maximum, one on the Pacific Coast and one east of the Rocky Mountains, and now remain distinct lineages with little evidence of gene flow between them. Southwestern British Columbia is a notable exception, as subspecies mixing between central British Columbia and Vancouver Island populations suggests a possible contact zone in this region. Z‐linked data support two mitochondrial groups; however, Coastal Oregon and central British Columbia sites show evidence of mixing. Contemporary population structure based on microsatellite data identified at least six genetic clusters: three *H. p. purpureus* clusters, two *H. p. californicus* clusters, and one mixed cluster, which likely resulted from high site fidelity and isolation by distance, combined with sexual selection on morphological characters reinforcing subspecies differences.

## 1 Introduction

Ancient vicariance events including geological processes such as plate tectonics, mountain formation, and glaciation have all influenced evolution (Apte, Smith, & Wallis, 2007; Brubaker, Anderson, Edwards, & Lozhkin, 2005). Glaciation has shaped the evolutionary history of many species, especially high‐latitude species, by creating fragmented populations that evolved independently of one another (Hofreiter et al., 2004; Walter & Epperson, 2005). During the last glacial maximum (LGM) 21,000 years ago, much of northern North America was under sheets of ice, forcing most organisms into glacial refugia where climatic conditions were amenable to survival. There is support for populations surviving in Pleistocene refugia in Beringia (Brubaker et al., 2005; Lait & Burg, 2013), off the coast of Newfoundland (Lait & Burg, 2013; Sonsthagen, Talbot, Scribner, & Mccracken, 2011), and in large areas within the contiguous United States and Mexico (Crespi, Rissler, & Browne, 2003; Grus, Graves, & Glenn, 2009). As the ice sheets retreated, populations expanded into previously glaciated habitat. Many contemporary population genetic patterns of temperate species are the result of postglacial dispersal and levels of population connectivity (Clark, Brown, Stechert, & Zamudio, 2010).

Barriers influencing population connectivity can be historical or contemporary, physical or nonphysical, and natural or manmade (Apte et al., 2007; Clark et al., 2010; Friesen, Burg, & McCoy, 2007; Qvarnström, Rice, & Ellegren, 2010). Evidence of natural, physical barriers restricting dispersal include the presence of unique genetic groups in populations separated by the Cabot Strait and the Strait of Belle Isle (Holder, Montgomerie, & Friesen, 2004; Lait & Burg, 2013). Not all barriers are large. Barriers can be small, such as roads (Clark et al., 2010) and agriculture fields (Bush et al., 2011). Behavior, including strong natal site fidelity, migratory routes, foraging sites, or mate choice (Friesen et al., 2007), can lead to reproductive isolation.

**Figure 1:**
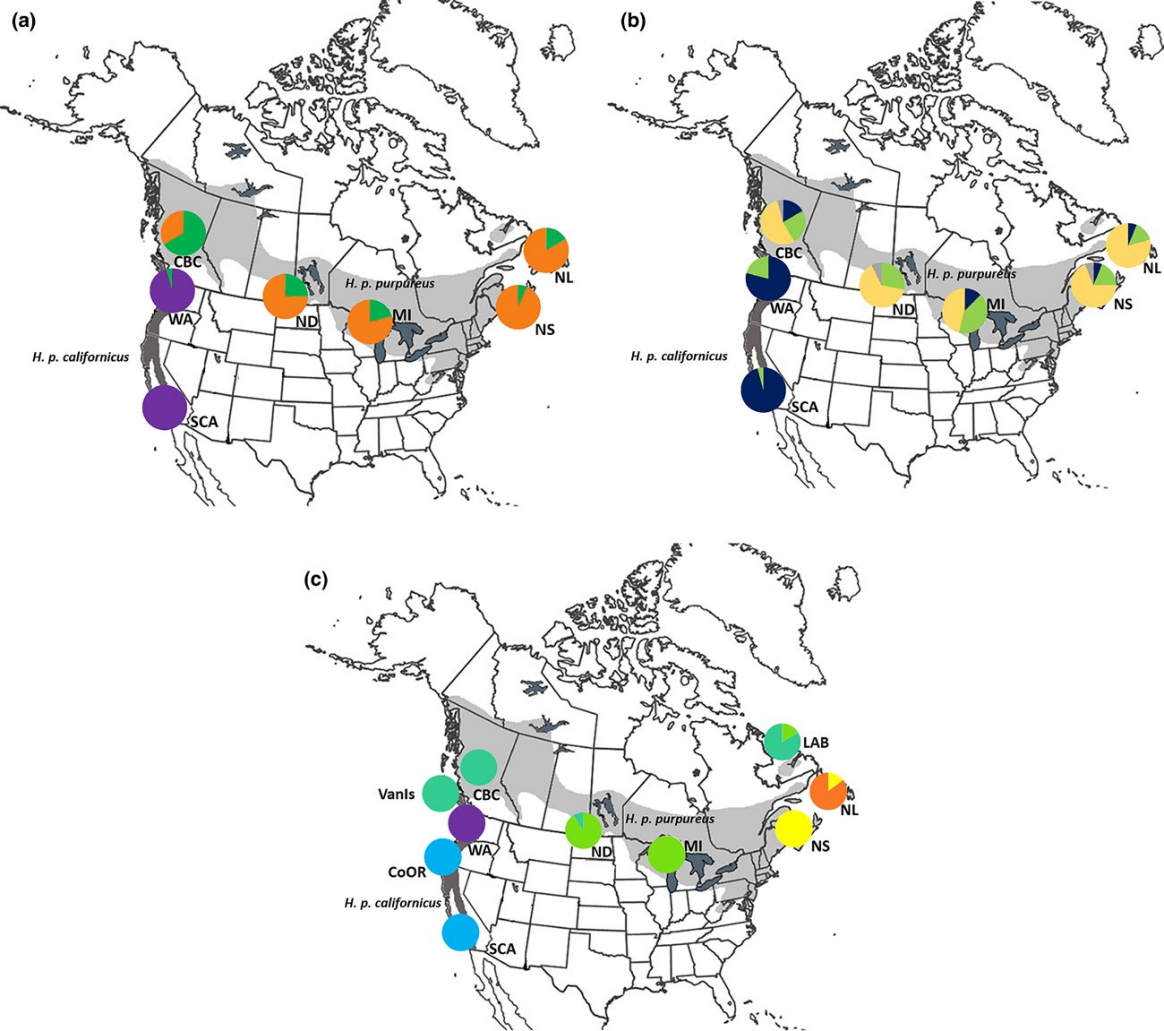
Assignment of individuals based on BAPS analysis of mtDNA (a) and z‐linked (b) loci and STRUCTURE analysis of microsatellite loci (c). Mitochondrial locus (a) shows *H. p. californicus* (purple) and *H. p. purpureus* clusters (orange and green). Z‐linked locus (b) shows four clusters corresponding to a predominantly *H. p. californicus* group (blue), a widespread (green), *H. p. purpureus* (yellow), and a cluster with no obvious geographic clustering (gray). Microsatellites (c) show two eastern maritime clusters (orange and yellow), a central continental cluster (green), a western *H. p. purpureus* and VanIs cluster (teal), two *H. p. californicus* clusters (purple and blue). Figure c includes all sampling sites. Map shading depicts the breeding ranges of *H. p. purpureus* (light grey) and *H. p. californicus* (dark grey)

The purple finch (*Haemorhous purpureus*) is a North American songbird with two recognized subspecies: *H. p. purpureus* to the east of the Rocky Mountains and *H. p. californicus* on the West Coast (Figure 1). The two subspecies exhibit morphological, plumage, vocal, and behavioral differences, in addition to variation within the subspecies (Wootton, 1996). The only genetic work conducted on the purple finch (Marten & Johnson, 1986) supports the current taxonomy, but is limited in both the number of sample sites (*n* = 2) and sample sizes (*n* = 2 and 15). Using a more representative sampling regime covering more of the purple finch's distribution, and several molecular makers, the aim of this study was to examine the evolutionary history and the effects of dispersal barriers on contemporary genetic patterns.

We examined three hypotheses related to purple finch evolution. First, we hypothesize that given their wide distribution across North America and large number of morphological differences between subspecies, the two subspecies of purple finch have been isolated for a prolonged period of time and likely survived the LGM in separate refugia. The Atlantic Shelf was a refugium for a number of species, including tree species upon which purple finch rely for food and nesting (Brubaker et al., 2005; Walter & Epperson, 2005). We predict the Atlantic Shelf and areas south of ice sheets as candidate refugia for the purple finch given their current distribution. Second, with the morphological and behavioral differences and relatively small contact zone between subspecies, we hypothesize no or low levels of gene flow between subspecies. We predict a large number of genetic differences between subspecies, and little haplotype/allele sharing. Third, based on findings from other studies, we hypothesize that contemporary barriers, that is, bodies of water and geographic distance, isolate *H. purpureus* populations. The straits separating both Newfoundland and Vancouver Island from the mainland correspond to patterns of genetic isolation in other taxa with similar dispersal mechanisms (Holder et al., 2004; Lait & Burg, 2013). We therefore predict populations on either side of these boundaries will be genetically distinct. Given the species large range, we predict isolation by distance. Lastly, we predict higher levels of population differentiation in the nonmigratory *H. p. californicus* than the migratory *H. p. purpureus*, as many sedentary species have been shown to exhibit low dispersal rates (Barrowclough, Groth, Mertz, & Gutierrez, 2004; Burg, Gaston, Winker, & Friesen, 2005; Spellman, Riddle, & Klicka, 2007).

## 2 Methods and Materials

### 2.1 Sample acquisition

Two hundred and sixty‐seven purple finches were sampled from 10 populations: Central British Columbia (CBC), Coastal Oregon (CoOR), Labrador (LAB), Michigan (MI), North Dakota (ND), Newfoundland (NL), Nova Scotia (NS), Southern California (SCA), Vancouver Island (VanIs), and Washington (WA) (Figure 1). Individuals were captured across their breeding range in May to July from 2007 to 2013 using 12‐m mist nets and playback recordings. Sampling was restricted to summer to reduce the number of migrants caught. A small (<50 μl) blood sample was taken from the brachial vein and the birds were banded and released on site. Blood was stored in 99% ethanol.

### 2.2 DNA extraction

Total genomic DNA was extracted from ~10 μl of blood using a modified Chelex extraction (Walsh, Metzger, & Higuchi, 1991). Following extraction, all samples were stored at −20°C.

### 2.3 Mitochondrial and Z chromosome DNA amplification and sequencing

A 707‐bp fragment of the ATP region of the mitochondrial genome, hereafter referred to as ATP6, containing both ATP 6 (271 bp) and ATP 8 (445 bp) was amplified using primers L8950 (CCAACCACAGCTTCATACCA) and H9694 (GCTAGTGGGCGGATGAGTAG) for 243 samples. The thermal cycling profile was one cycle of 120 s at 94°C, 45 s at 54°C, 60 s at 72°C; 37 cycles of 30 s at 94°C, 45 s at 54°C, and 60 s at 72°C; and a final cycle of 5 min at 72°C. The 25 μl PCR contained: Green GoTaq^®^ Flexi buffer (Promega), 0.2 mmol/L dNTP, 2.5 mmol/L MgCl_2_, 0.4 μmol/L each primer, 1 U GoTaq^®^ Flexi polymerase, and genomic DNA.

A 515‐bp fragment of the AldB region of the Z chromosome was amplified in 178 samples for a total of 306 alleles, using primers AldB6‐F and AldB8‐R (Hackett et al., 2008). This region on the Z chromosome was amplified using a similar thermal cycling program as the ATP6 reactions with the exception of a 50°C annealing temperature, 2.0 mmol/L MgCl_2_, and crimson buffer (New England Biolabs).

Successfully amplified samples were sent to NanuQ sequencing service at McGill University, Montreal, Quebec. Sequences were checked and aligned using MEGA v. 5 (Tamura et al., 2011).

### 2.4 Microsatellite amplification

Three *H. purpureus* samples from CBC, NL, and VanIs were used to test 39 microsatellite loci designed from other avian species. Nine loci which worked consistently were screened for polymorphism with another 12 individuals from across the range. A total of seven loci were polymorphic and used to screen 260 samples. Six of the polymorphic loci: CETC 215 (Poláková et al., 2007), Ase18 (Richardson, Jury, Dawson, & Salgueiro, 2000), PAT MP 43 (Otter, Ratcliffe, Michaud, & Boag, 1998), Titgata 39, Titgata 02 (Wang, Hsu, Te, & Li, 2005), and CtC 101 (Tarvin, 2006) were amplified using one cycle of 120 s at 94°C, 45 s at 50°C, and 60 s at 72°C; seven cycles of 60 s at 94°C, 30 s at 50°C, and 45 s at 72°C; 25 cycles of 30 s at 94°C, 30 s at 52°C, and 45 s at 72°C; and a final cycle of five minutes at 72°C. A seventh locus, Cuu28 (Gibbs, Tabak, & Hobson, 1999), was amplified with the same program, except annealing *T*_m1_ and *T*_m2_ were 45 and 48°C, respectively. The 10 μl PCR contained: 0.2 mmol/L dNTP, 2 mmol/L MgCl_2_ (2.5 mmol/L for CtC 101 and CETC 215, and 1.5 mmol/L for PAT MP 43), 1 μmol/L each primer, 0.5 U of taq polymerase, and 0.05 μmol/L fluorescently labeled M13 tag.

### 2.5 Genetic analyses

#### 2.5.1 Sequence data

AldB sequences from all known male birds were run through PHASE v. 2.0 (Stephens & Donnelly, 2003) in DnaSP v. 5.1 (Librado & Rozas, 2009) to account for the presence of two Z chromosome sequences in males. Four individuals from NS were removed due to highly dissimilar z‐linked sequences, although their mitochondrial sequences matched other purple finches. Haplotype (h) and nucleotide diversity (π) indices were calculated in DnaSP 5.1 (Librado & Rozas, 2009). We calculated a time of divergence between the two subspecies using a divergence rate at the cytochrome b locus of mtDNA of 2.2% nucleotide differences per site per million years based on work by Fleischer et al. (1998) on Hawaiian honeycreepers.

Genetic differentiation between sampling sites was examined using *F*_ST_ values calculated in genalex v. 6.5 (Peakall & Smouse, 2006). Due to small sample size (*n* ≤ 6), LAB, CoOR, and VanIs sites were removed from all pairwise *F*_ST_ analysis. *p*‐values were corrected using the modified false discovery rate (FDR) (Benjamini & Hochberg, 1995).

AMOVAs were performed in Arlequin v. 3.5.1.3 to test for the diversity found between the two subspecies, among the populations, and within the populations (Excoffier & Lischer, 2010). Principal coordinate analysis (PCoA) on Φ_ST_ and *F*_ST_ values in genalex for mitochondrial DNA and z‐linked loci, respectively, allowed us to quantify the variance in our data.

A Bayesian clustering program, BAPS v. 5.2, was used to group individuals as BAPS forms clusters with no a priori defined populations. The analysis was run with the linked loci option (Corander, Waldmann, & Sillanpää, 2003).

The relationships between all haplotypes were examined using statistical parsimony networks created in TCS v. 1.21 (Clement, Posada, & Crandall, 2000) for both mtDNA and z‐linked loci. All connections were made with a 95% connection limit.

#### 2.5.2 Microsatellite data

Amplified DNA was run on an acrylamide gel with a ladder and controls on the NEN Model 4300 DNA Analyzer (Licor BioSciences). Individuals missing more than three of the seven loci (*n* = 3) were removed from the analyses. A total of 260 individuals from all 10 populations were successfully genotyped at the seven loci. Populations and loci were checked for deviations from Hardy–Weinberg equilibrium and linkage disequilibrium with GENEPOP (Rousset, 2008), and evidence of large allele dropout, null alleles, and genotyping errors were examined using Microchecker (Van Oosterhout, Hutchinson, Wills, & Shipley, 2004).

Genotype data were run through the Bayesian clustering program STRUCTURE v. 2.3.4 to determine the number of clusters found in the data (Pritchard, Stephens, & Donnelly, 2000). Because this type of analysis uses individuals and is not as sensitive to levels of sampling within populations, LAB, CoOR, and VanIs were included. A burn‐in of 50,000 and MCMC length of 150,000 were used with admixture model and loc priors. Ten iterations were performed for each cluster (K) 1‐10. Structure Harvester v. 0.6.94 (Earl & vonHoldt, 2012) was used to calculate average likelihoods for each value of K and implement the Evanno method (Evanno, Regnaut, & Goudet, 2005). ∆K and Ln P(D) were used to determine the optimal number of clusters present. As suggested by Pritchard et al. (2000), a hierarchical analysis was performed by rerunning the analysis for each cluster to test for further structure.

In addition to conducting cluster analysis, genetic differentiation between populations was calculated using Wright's *F*_ST_ index (Wright, 1965) and Hedrick's *G′*_ST_ (Hedrick & Goodnight, 2005) in genalex v. 6.5 (Peakall & Smouse, 2006). Using the *F*_ST_ values, an AMOVA and a principal coordinate analysis were conducted in Arlequin v. 3.5 (Excoffier & Lischer, 2010).

Discovery of potential barriers was conducted using Barrier v. 2.2 (Manni, Guérard, & Heyer, 2004), which uses geographic information and genetic distance information (pairwise *F*_ST_ matrices) to map putative barriers according to Monmonier's algorithm (Monmonier, 1973).

### 2.6 Isolation by distance

To clarify whether genetic differentiation is a result of geographic distance, Mantel tests were performed comparing genetic and geographic distances to test for isolation by distance. Tests were completed for all three datasets in genalex v. 6.5 using shortest geographic distances between sampling sites through suitable forested habitat.

### 2.7 Spatio‐geographic modeling

Spatio‐geographic modeling was used to identify potential Pleistocene refugia. Occurrence information for the purple finch (>500,000 occurrences) was obtained from the Global Biodiversity Information Facility. After removing points with no UTMs, potential outliers (outside the expected range), duplicated data, samples from migratory periods, or any data from potentially unreliable sources, 5,710 points remained. Five of the 19 climate layers with ≥0.9 threshold in ENMTools (Warren, Glor, & Turelli, 2008) were used. The climatic layers included maximum temperature of warmest month, mean temperature of warmest quarter, mean temperature of coldest quarter, precipitation of wettest month, and precipitation of driest month. A file containing the UTMs of the 5,710 purple finch locations was loaded into MaxEnt v. 3.3.3 (Phillips, Anderson, & Schapire, 2006), along with the five Bioclim climatic layers. MaxEnt was run with the recommended hinge only function to reduce model overfit. Ten replicates were run with the cross‐validate function. The migratory *H. p. purpureus* and the resident *H. p. californicus* were also run separately in case their separate distributions influenced the models, but these did not change the projections and therefore were not included. MaxEnt was then used to model possible distributions during the LGM (21,000 years ago) and LIG (120,000–140,000 years ago).

## 3 Results

### 3.1 Sequence data

#### 3.1.1 ATP6 sequence data

Diversity indices for the ATP6 locus varied. Excluding populations with fewer than four individuals, haplotype diversity ranged from 0.270 (SCA) to 0.899 (ND), and nucleotide diversity ranging from 0.0004 (SCA) to 0.0056 (NS) (Table 1). The average nucleotide difference between subspecies was 2.4%. A coalescence time of 1.1 MYA was calculated using the fixed differences in the mitochondrial dataset between the two subspecies.

**Table 1:** Haplotype (h) and nucleotide (π) diversity values, sample size (*n*), number of haplotypes (Nh) in each population at the mitochondrial and z‐linked loci

Pop	mtDNA ATP				Z chromosome AldB				Microsatellite			
	*h*	π	*n*	Nh	*h*	π	*n*	Nh	*H*_0_ ± *SE*	*H*_E_ ± *SE*	*A*_R_ ± *SE*	*n*
VanIs	0.000	0.0000	4	1	0.000	0.0000	4	1	–	–	–	
CoOR	1.000	0.0029	2	2	0.786	0.0062	8	4	–	–	–	
WA	0.679	0.0015	38	10	0.486	0.0017	39	14	0.506 ± 0.092	0.592 ± 0.119	4.881 ± 1.411	39
SCA	0.270	0.0004	28	5	0.086	0.0005	46	3	0.528 ± 0.148	0.498 ± 0.13	4.494 ± 1.367	30
NL	0.889	0.0026	34	16	0.464	0.0024	50	6	0.514 ± 0.128	0.572 ± 0.109	5.687 ± 1.204	33
CBC	0.858	0.0024	53	17	0.816	0.0052	56	16	0.492 ± 0.137	0.529 ± 0.142	6.517 ± 1.470	59
NS	0.857	0.0056	35	14	0.604	0.0038	47	6	0.587 ± 0.116	0.659 ± 0.093	6.015 ± 1.067	42
ND	0.899	0.0028	24	14	0.837	0.0069	26	12	0.431 ± 0.152	0.467 ± 0.139	4.801 ± 1.298	22
MI	0.661	0.0018	19	7	0.754	0.0045	26	12	0.352 ± 0.141	0.417 ± 0.129	6.583 ± 1.457	20
LAB	0.733	0.0014	6	3	0.500	0.0058	4	2	–	–	–	

Mean observed heterozygosity (*H*_0_), expected heterozygosity (*H*_E_), allelic richness (*A*_R_), and standard errors (*SE*) for microsatellite data.

**Figure 2:**
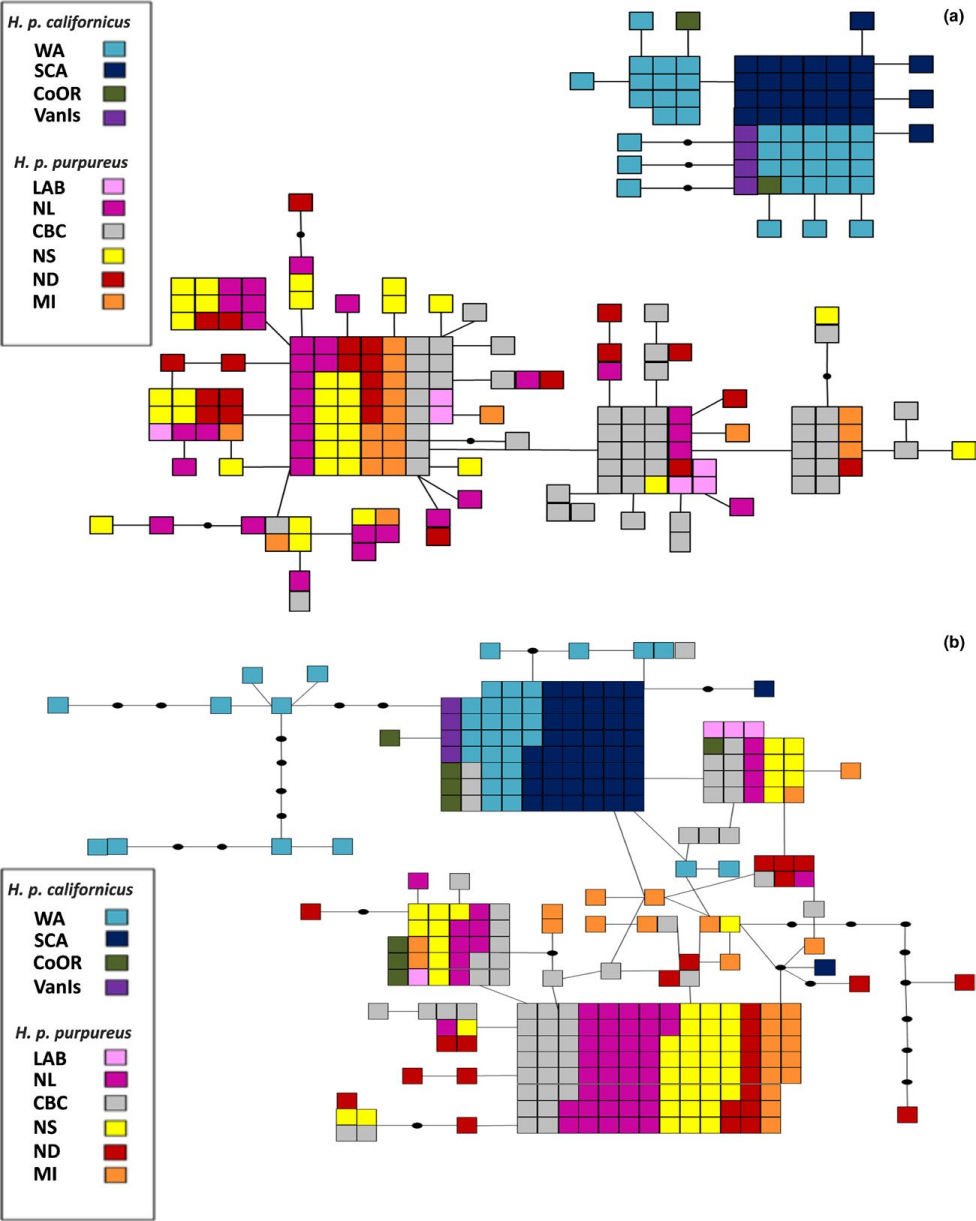
Parsimony haplotype networks from the mitochondrial (a) and z‐linked (b) loci. Each square represents an individual and a group of squares represents individuals that share a haplotype, lines connecting squares represent a single nucleotide difference, and black dots are unsampled, or inferred haplotypes (see legend for population color‐coding). *H. p. californicus* and *H. p. purpureus* individuals are represented by cool and warm colors, respectively, with the exception of *H. p. purpureus* individuals from CBC, which are in gray

The ATP6 parsimony haplotype network contained a total of 56 haplotypes. Nineteen of the haplotypes were found in more than one individual and four of these were restricted to a single population. Of the 56 haplotypes, 44 were found in a single individual or population (Table 3a). *H. p. californicus* and *H. p. purpureus* formed two distinct clusters with no haplotype sharing (Figure 2a). *H. p. californicus* had two shared and 13 single haplotypes while *H. p. purpureus* had 17 shared and 24 single haplotypes.

Pairwise Φ_ST_ values ranged from 0.000 to 0.960 with the highest Φ_ST_ value between MI and SCA from different subspecies (Table 2a). Between the two *H. p. californicus* populations (WA and SCA), Φ_ST_ was 0.092, while for the five *H. p. purpureus* populations, Φ_ST_ values ranged from 0.000 to 0.194. Average Φ_ST_ between subspecies was 0.9193. All pairwise comparisons between subspecies were significant and CBC was significantly different from all other *H. p. purpureus* populations and the two *H. p. californicus* populations were significantly different from each other.

**Table 2:** Mitochondrial (ATP6) pairwise Φ_ST_ and z‐linked (AldB) pairwise *F*_ST_ comparisons (below diagonal) and corresponding significance scores (above diagonal). Microsatellite pairwise F_ST_ and G’_ST_ (italicized) comparisons (below diagonal) and corresponding P‐values (above diagonal). Values in bold remained significant following FDR corrections

		WA	SCA	NL	CBC	NS	ND	MI
(a) ATP6								
*H. p. californicus*	WA	*	**<0.0001**	**<0.0001**	**<0.0001**	**<0.0001**	**<0.0001**	**<0.0001**
	SCA	0.092	*	**<0.0001**	**<0.0001**	**<0.0001**	**<0.0001**	**<0.0001**
*H. p. purpureus*	NL	0.898	0.932	*	**<0.0001**	0.820	0.099	0.306
	CBC	0.903	0.928	0.180	*	**<0.0001**	**<0.0001**	**<0.0001**
	NS	0.898	0.932	0.000	0.194	*	0.060	0.306
	ND	0.896	0.936	0.019	0.131	0.017	*	0.234
	MI	0.910	0.960	0.005	0.113	0.006	0.014	*
(b) AldB								
*H. p. californicus*	WA	*	**<0.0001**	**<0.0001**	**<0.0001**	**<0.0001**	**<0.0001**	**<0.0001**
	SCA	0.116	*	**<0.0001**	**<0.0001**	**<0.0001**	**<0.0001**	**<0.0001**
*H. p. purpureus*	NL	0.621	0.805	*	**0.009**	0.405	0.054	0.099
	CBC	0.438	0.558	0.060	*	0.189	0.135	0.847
	NS	0.535	0.707	0.000	0.007	*	0.144	0.522
	ND	0.462	0.649	0.048	0.020	0.017	*	0.414
	MI	0.467	0.695	0.034	0.000	0.000	0.000	*
(c) Microsatellites								
*H. p. californicus*	WA	*	**<0.0001/** ***0.014***	**<0.0001/** ***0.014***	**<0.0001/** ***0.014***	**<0.0001/** ***0.014***	**<0.0001/** ***0.014***	**<0.0001/** ***0.014***
	SCA	0.066/*0.105*	*	**<0.0001/** ***0.014***	**<0.0001/** ***0.014***	**<0.0001/** ***0.014***	**<0.0001/** ***0.014***	**<0.0001/** ***0.014***
*H. p. purpureus*	NL	0.095/*0.131*	0.080/*0.211*	*	**<0.0001/** ***0.014***	**<0.0001/** ***0.014***	**<0.0001/** ***0.014***	**<0.0001/** ***0.014***
	CBC	0.045/*0.050*	0.028/*0.081*	0.053/*0.115*	*	**<0.0001/** ***0.014***	**<0.0001/** ***0.014***	**<0.0001/** ***0.014***
	NS	0.109/*0.145*	0.083/*0.203*	0.076/*0.155*	0.049/*0.098*	*	**<0.0001/** ***0.014***	**<0.0001/** ***0.014***
	ND	0.070/*0.070*	0.047/*0.167*	0.065/*0.113*	0.045/*0.055*	0.09/*0.186*	*	**0.003/** ***0.014***
	MI	0.174/*0.213*	0.13/*0.384*	0.137/*0.264*	0.125/*0.223*	0.14/*0.327*	0.029/*0.082*	*

Significance values have been corrected for multiple comparisons using the modified FDR and significant comparisons are bolded. Microsatellite pairwise *F*_ST_ and *G′*_ST_ (italicized) comparisons (below diagonal) and corresponding *p*‐values (above diagonal). Values in bold remained significant following FDR corrections.

The significant differentiation between subspecies was supported by AMOVAs. The majority of the variance for the mitochondrial locus was explained by genetic differentiation between subspecies variation (90%), while 9% was explained by differences between individuals, and just 1% of the variation between populations.

**Figure 3:**
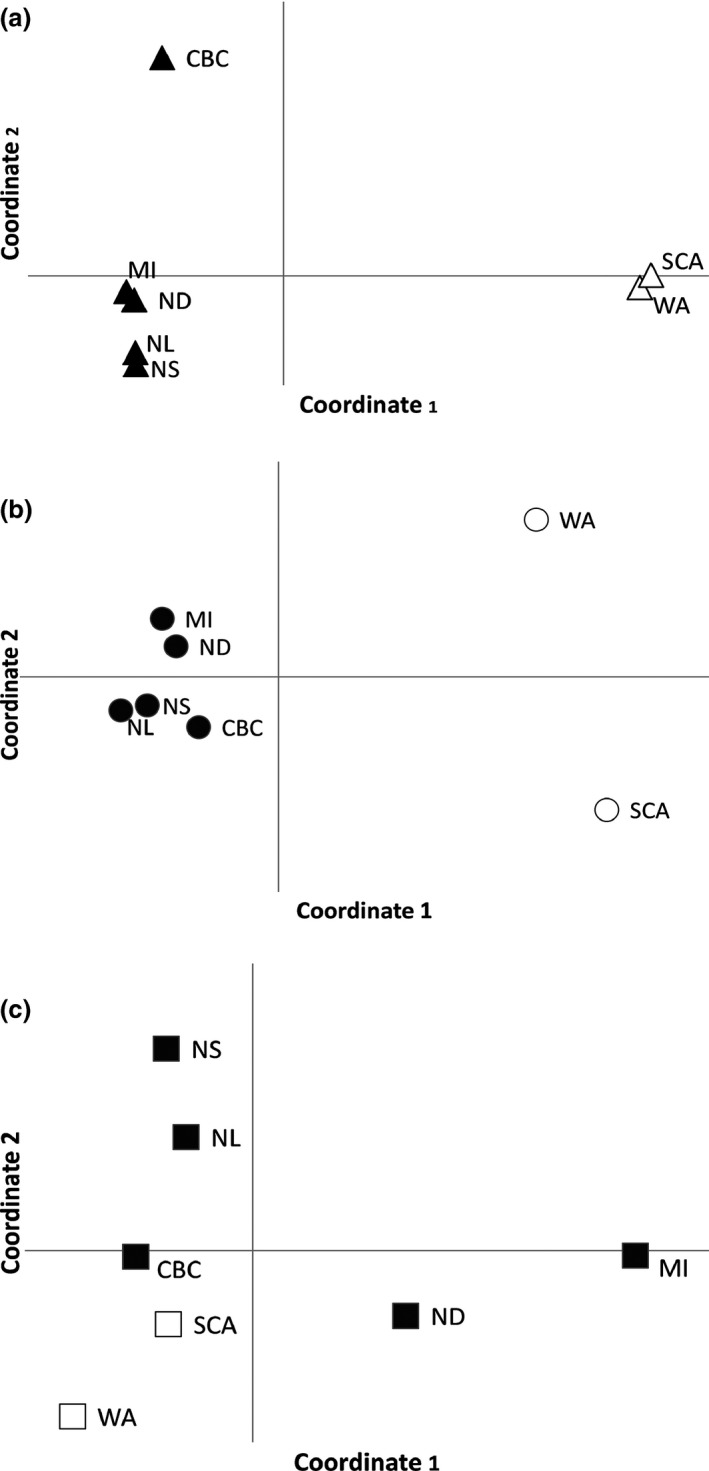
Principal coordinate analysis of the mtDNA (a), z‐linked (b), and microsatellite (c) loci. Coordinates 1 and 2 explain 87% and 9% of the mtDNA, 90% and 7% of the z‐linked, and 45% and 25% of the microsatellite variation, respectively. Black shapes represent *H. p. purpureus*, and white shapes represent *H. p. californicus*

Principal coordinate analysis for ATP6 showed coordinate 1 explained most of the variation (87.3%), while coordinates 2 (8.9%) and 3 (3.2%) explained the rest. The first two coordinates explained 96% of the variation and clustered geographically proximal populations together (Figure 3). BAPS identified three clusters for the mitochondrial locus, one *H. p. californicus* cluster and two predominantly *H. p. purpureus* clusters (Figure 1a). The two *H. p. purpureus* clusters showed an east–west gradient with one cluster predominantly occurring in the west and decreasing in frequency to the east, and an eastern cluster that is less common in the west.

#### 3.1.2 AldB sequence data

The final AldB dataset contained 306 sequences. The z‐linked chromosome showed lower diversity values than the mitochondrial ATP6, with a range of haplotype diversity values for populations of *n* > 4 from 0.086 in SCA to 0.837 for ND. Nucleotide diversity ranged from 0.0005 in SCA to 0.0069 in ND (Table 1). CBC had one of the highest haplotype diversities (*h* = 0.816) (Table 1).

The z‐linked locus shows more haplotype sharing between the two subspecies, but generally separates the subspecies, with the exception of four CoOR, one SCA, and four CBC individuals (Figure 2b). A total of 47 haplotypes were found at this locus. Thirty‐six haplotypes were restricted to one sampling site, including 33 that were found in a single individual (Table 3). CBC had the highest overall number of haplotypes (16) and the highest number of shared haplotypes (11) (Table 3).

**Table 3:** List of all haplotypes, broken down by population for the mitochondrial locus ATP6 (a) and z‐linked locus AldB (b) Unique haplotypes are found in one individual, private haplotypes are found in a single population

Haplotype	VanIs	CoOR	SCA	WA	CBC	ND	MI	NS	LAB	NL	Total
(a) ATP6											
A				11							**11**
B	4	1	24	19							**48**
C					14	1		1	3	3	**22**
D					10	1	3				**14**
E					2	1					**3**
F					2						**2**
G					3						**3**
H					1		1	2		1	**5**
I					1			1			**2**
J					11	7	11	12	2	10	**53**
K						4	1	4	1	2	**12**
L						1				1	**2**
M						2		5		5	**12**
*N*							1	1		3	**5**
O								2		1	**3**
P					1	1				1	**3**
Q					1					1	**2**
R						1				1	**2**
S								2			**2**
#Unique	**0**	**1**	**4**	**8**	**7**	**5**	**2**	**5**	**0**	**5**	**37**
#Private	**0**	**1**	**4**	**9**	**9**	**5**	**2**	**6**	**0**	**5**	**41**
#Haplotypes	**1**	**2**	**5**	**10**	**17**	**14**	**7**	**14**	**3**	**16**	**56**
Sample size	**4**	**2**	**28**	**38**	**53**	**24**	**19**	**35**	**6**	**34**	**243**
(b) z‐Linked locus AldB											
A	4	3	44	24	3						**78**
B		3			7		2	8	1	7	**28**
C		1			7		1	7	3	4	**23**
D					22	10	13	28		36	**109**
E							2				**2**
F					1	4				1	**6**
G					2	1		2			**5**
H					3	2		1		1	**7**
I					1	2					**3**
J					3						**3**
K							1	1			**2**
L					1		1				**2**
M				2	1						**3**
*N*				2							**2**
#Unique	**0**	**1**	**2**	**11**	**5**	**7**	**6**	**0**	**0**	**1**	**33**
#Private	**0**	**1**	**2**	**12**	**6**	**7**	**7**	**0**	**0**	**1**	**36**
#Haplotypes	**1**	**4**	**3**	**14**	**16**	**12**	**12**	**6**	**2**	**6**	**47**
Sample size	**4**	**8**	**46**	**39**	**56**	**26**	**26**	**47**	**4**	**50**	**306**

*F*_ST_ values for the z‐linked locus ranged from 0.000 to 0.805 (Table 2). The *F*_ST_ values were lower within species (0.116 in *H. p. californicus* and 0‐0.048 in *H. p. purpureus*) than between subspecies (0.438–0.805). All pairwise *F*_ST_ values between subspecies and between the two *H. p. californicus* populations were significant after corrections for multiple tests. The only *H. p. purpureus* comparison that was significant was NL–CBC.

The AMOVA from AldB showed 55%, 43%, and 2% of the variation is explained by differences between subspecies, within populations, and among populations, respectively. For AldB, PCoA coordinates 1 and 2 explained 90% and 7% of the variation (Figure 3) and populations clustered according to subspecies. BAPS found a total of four clusters and assigned at least one individual in all but one *H. p. purpureus* population to the *H. p. californicus* cluster and several *H. p. californicus* to a widespread cluster (Figure 1b).

#### 3.1.3 Genotype data

None of the seven microsatellite loci showed significant effects of large allele dropout or stuttering causing scoring errors for any of the populations. However, CETC 215 showed a homozygote excess, suggesting null alleles in four of the populations (NS, NL, CBC, and SCA), while Microchecker suggested possible null alleles for Titgata 39 with NL and ND samples, for Titgata 02 among NL and CBC samples, and Ase18 among CBC samples. Analyses were run with and without CETC 215; however, the results were not different so CETC 215 was kept in the analysis.

Mean observed heterozygosities ranged from 0.352 (MI) to 0.587 (NS), and mean allelic richness ranged from 4.494 (SCA) to 6.583 (MI) (Table 1). Population pairwise *F*_ST_ comparisons ranged from 0.028 to 0.174, with all 19 comparisons significant (Table 2c). The average *F*_ST_ values between subspecies and within *H. p. purpureus* were 0.0861 and 0.0809, respectively. AMOVA revealed that the majority of variance found is due to variation among populations (92%), while 8% explained variation between populations, and there was a negligible contribution of variation from comparisons between regions (subspecies) at 0.5%.

Coordinates 1 and 2 explained a total of 70% of the variation (45% and 25%, respectively), while coordinate 3 explained an additional 16% (Figure 3c). Unlike the mtDNA and z‐linked loci, there is no obvious clustering in the PCoA (Figure 3c). Coordinate 1 separates central populations (ND and MI) from the other populations, and coordinate 2 separates eastern (NS and NL) from western (SCA, CBC, and WA) and central populations. The CBC *H. p. purpureus* population clusters closer to *H. p. californicus*.

**Figure 4:**
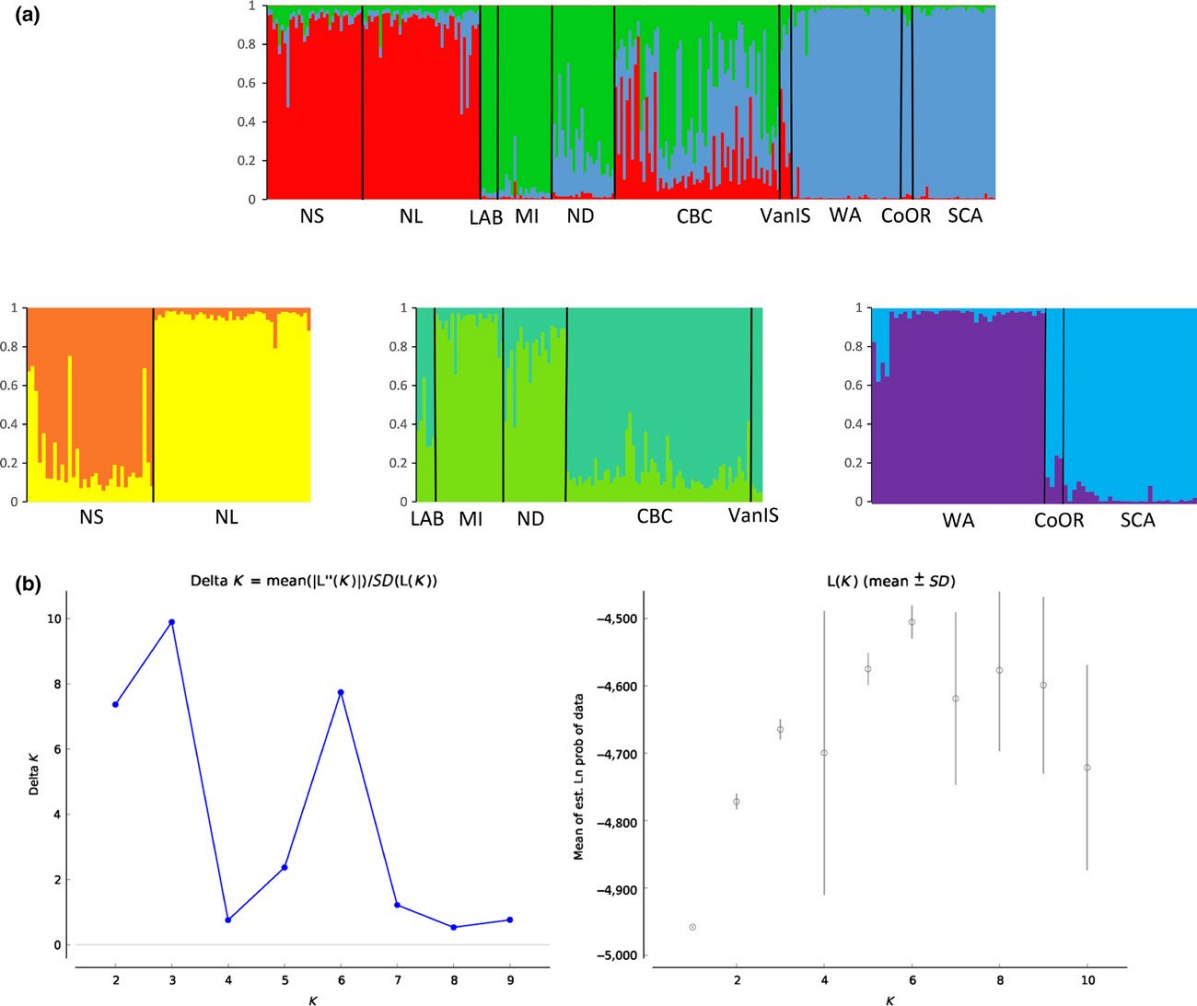
Hierarchical runs from STRUCTURE (a). After the initial run at K = 3 (top plot), clusters were separated to examine additional structure (bottom plot, K = 6). Statistical output from Structure Harvester (b) suggesting optimal K with ΔK and Ln P(D)

STRUCTURE and Structure Harvester revealed three clusters with the Evanno method and six clusters using Bayes’ rule (Pritchard et al., 2000) (Figure 4b). The three clusters separated eastern populations (NL and NS), central populations (LAB, MI, and ND), and western populations (WA, CoOR, SCA) with CBC and VanIs showing mixing between these groups. Hierarchical analysis for each of these three clusters divided the clusters further for a total of six groups: (i) NL, (ii) NS, (iii) LAB, MI, and ND, (iv) CBC and VanIs, (v) WA, and (vi) CoOr and SCA. CBC individuals showed mixed ancestry, assigning to the central populations of ND and MI, and the CBC and VanIs cluster (Figures 3c, 4). BARRIER 2.2 discovered a single barrier between NS and MI separating eastern populations of LAB, NS, and NL from the other populations.

### 3.2 Isolation by distance

Isolation by distance was significant using mtDNA (*r* ^2^ = .261, *p* = .003) and z‐linked data (*r* ^2^ = .387, *p* = .001). However, it was not significant (*r* ^2^ = .018, *p* = .101) for microsatellite data.

### 3.3 Spatio‐geographic modeling

The model resulting from running nine climatic layers and over 5,710 occurrence locations performed significantly better than random. The average omission rate for this model closely resembled the predicted omission rate, meaning few occurrences were in unsuitable areas. The area under the curve (AUC) was 0.944 (±0.005), where 0.5 is random and values closer to one denote a greater ability to distinguish between suitable and unsuitable habitat (Carstens & Richards, 2007).

**Figure 5:**
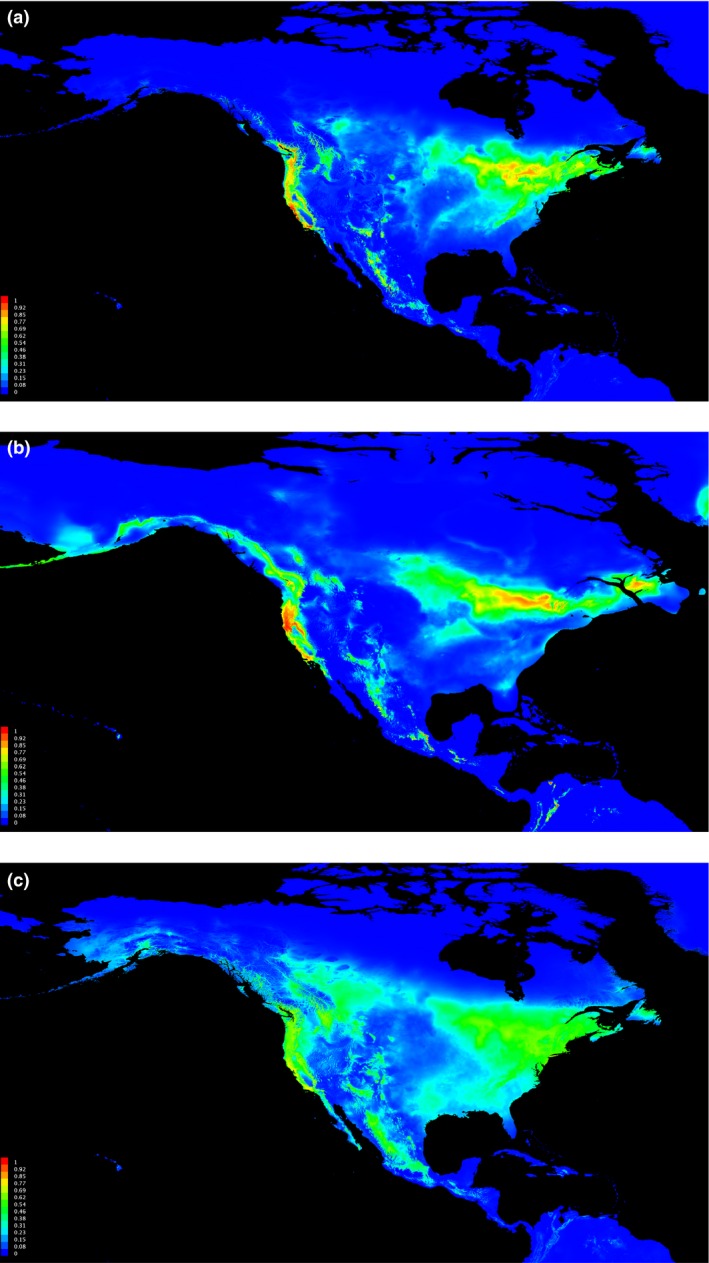
Projection of suitable climatic conditions for purple finch in the present day (a), from the LGM (~21,000 years ago) (b), and from LIG (~120,000–140,000 years ago) (c). Areas of suitable conditions in red are based on trained data from 2,710 presence occurrences, five uncorrelated climate layers, and the MIROC‐LJP Global Climate Model

The predicted distribution of *H. p. purpureus* closely resembled the actual distribution (Figure 5a) (Sibley, 2011). Bioclim layer 10 (mean temperature of warmest quarter) and 11 (mean temperature of coldest quarter) explained 30.2% and 25.3%, respectively, of the distribution of the purple finch. When the LGM layers were used on trained data, the model highlighted four large areas of suitable habitat (Figure 5b). The Pacific Coast shows suitable purple finch habitat, as do two large areas along the present‐day Canadian/USA border in the Midwest along the southern extent of the ice sheets, and further west in current‐day Arizona and Mexico. In addition to the large potential refugia south of the ice sheets, MaxEnt identified suitable habitat off the coast of Newfoundland. LIG projections showed isolation between eastern and western areas of suitable habitat (Figure 5c).

## 4 Discussion

### 4.1 Multiple refugia

Our results, particularly the AldB and ATP6 loci, provided support for the hypothesis that the two subspecies likely experienced prolonged isolation after a Pleistocene era split approximately 1.1 MYA. *H. purpureus* survived in two different refugia: *H. p. californicus* in one and *H. p. purpureus* in another (Figure 5b). The ENM shows four locations with suitable habitat during the LGM: one within much of the current *H. p. californicus* range on the Pacific Coast, another to the east of the Sierra Nevada Mountains, a third large area south of the ice sheets in the east, and a fourth off the Grand Banks of Newfoundland (Figure 5b).

The current breeding distribution of *H. p. purpureus* lies within previously glaciated areas. This suggests a refugium outside of the current distribution for *H. p. purpureus*, and it possibly persisted in a single refugial area in the southeastern USA (Figure 5). *H. p. purpureus* mtDNA haplotypes, which retain signatures of historical genetic structuring, are distributed across the range and are not divergent (Figures 1a, 2a, 3a), suggestive of expansion from a single refugium. This is supported by the large number of shared haplotypes, pairwise Φ_ST_ (except CBC), low among population variation of 1% in mtDNA, and similar haplotype diversity values among populations (Table 1).

ATP and AldB data give a clearer picture of historic distributions of *H. p. californicus*. The lower genetic diversity in SCA and the high nucleotide and haplotype diversity values in WA (Table 1) suggest the *H. p. californicus* refugium was in the northern part of the current range. Diversity values in WA are more congruent with an older population which supports the idea that *H. p. californicus* survived the LGM in a northwestern refugium (Figure 5). Previous studies have purported Pacific Northwest refugia for a number of diverse taxa (Byun, Koop, & Reimchen, 1997; Soltis, Gitzendanner, Strenge, & Soltis, 1997).

The nucleotide and haplotype diversity values of *H. p. californicus* suggest a southward expansion of *H. p. californicus* into SCA. The SCA population has the lowest haplotype and nucleotide diversity values of all the populations with the exception of the undersampled VanIs (Table 1). Low diversity indices can result from bottlenecks or founder effects, and are more common along the periphery of a species range than in the center (Bush et al., 2011; Grus et al., 2009).

### 4.2 Contemporary structure

*H. p. purpureus* and *H. p. californicus* populations were able to maintain strong genetic differentiation after postglacial expansion, as evidenced by mtDNA and z‐linked loci at many metrics (pairwise Φ_ST_/*F*_ST_ comparisons, AMOVA, PCoA, and BAPS). The mtDNA haplotypes show separation of *H. p. purpureus* populations from *H. p. californicus* (Figure 2). Microsatellite data indicate contemporary structure with significant population genetic differentiation (Table 2) among six isolated groups (Figure 4a). One cluster containing CBC and VanIs grouped both subspecies. While this may be influenced by small population size for VanIs, AldB also suggests mixing in those populations. While mitonuclear discordance is often evidence of incomplete lineage sorting, this phenomenon is not expected to produce the geographic patterns of differentiation seen in our data.

The contemporary mixing between the two subspecies in CBC and VanIs could result from one of three factors. The high genetic diversity (Table 1) in CBC might indicate a separate evolutionary history from other *H. p. purpureus* populations, but secondary contact can also cause high diversity values. Given CBC's proximity to a potential contact zone with *H. p. californicus* populations, secondary contact may be a more likely explanation. Admixture in the AldB locus suggests limited secondary contact, for which there is regional evidence in Townsend's and black‐throated green warblers (*Dendroica townsendi*,* D*. virens) and Swainson's thrushes (*Catharus ustulatus*) (Ruegg, 2007; Toews, Brelsford, & Irwin, 2011). The considerable geographic sampling gap between CBC and the closest sampled *H. p. purpureus* population in ND may exaggerate the genetic isolation of CBC from other *H. p. purpureus* populations, especially given the significant IBD. Lastly, differing migratory behaviors could explain the discrepancy between CBC and other *H. p. purpureus* birds. CBC birds overwinter in the southwestern United States, while the other populations of *H. p. purpureus* that we sampled migrate south to the east of the Rocky Mountains (Wootton, 1996).

STRUCTURE showed mixing among several sampling sites. Interestingly, VanIs (*H. p. californicus*) individuals clustered with *H. p. purpureus* birds from CBC (Figures 3c, 4), and the CBC population clustered separately from other *H. p. purpureus* populations (Figures 3c, 4). CBC and VanIs populations, while from different subspecies, are geographically close in proximity. The sample size for the VanIs population is low (*n* = 4), three individuals from the well‐sampled CBC population shared a haplotype with the VanIs population at the z‐linked locus, and another CBC bird grouped with two WA individuals (Figure 2b). The mixing of CBC and VanIs populations is plausible, and further evidence that a *H. p. purpureus*/*H. p. californicus* contact zone may exist in southwestern BC.

Mixing is also evident in other populations. In SCA and CoOR, we caught three individuals with *H. p. purpureus* z‐haplotypes and *H. p. californicus* mtDNA haplotypes (Figure 2a,b) amounting to four alleles, one from a SCA female and three from two CoOR males. Despite the fact that *H. p. purpureus* overwinter in *H. p. californicus* range, migration routes do not explain the presence of *H. p. purpureus* z‐haplotypes within the *H. p. californicus* range as *H. p. purpureus* migrants would not have *H. p. californicus* mtDNA. We also found CBC *H. p. purpureus* with *H. p. californicus* z‐linked alleles and *H. p. purpureus* mtDNA (three individuals, four alleles). The presence of *H. p. purpureus* nuclear DNA within the *H. p. californicus* range and *H. p. californicus* nuclear DNA in the *H. p. purpureus* range, along with the clustering of CBC and VanIs populations, suggests limited gene flow between the two subspecies at some western sites. This could occur through hybridization at a contact zone with male‐biased dispersal as the pattern is only seen in nuclear loci. Similar patterns of male gene flow are found in the spectacled eider (*Somateria fischeri*) (Scribner et al., 2001). Secondary contact between groups from separate glacial refugia has been suggested for several species across multiple taxa in western Canada, including mountain goats (*Oreamnos americanus)*, boreal chickadees (*Poecile hudsonicus*), and Sitka and white spruce (*Picea sitchensis* and *P. glauca*) (Hamilton & Aitken, 2013; Lait & Burg, 2013; Shafer, Côté, & Coltman, 2011).

### 4.3 Barriers

Both physical and nonphysical barriers correspond to contemporary population genetic patterns in *H. purpureus*. Behavioral differences including migration and site fidelity, and differences in plumage correspond to genetic patterns. Migration behavior can explain the high genetic diversity between subspecies. With the exception of the aforementioned potential mixing in a small number of individuals in the Pacific Northwest, both resident and migratory populations show significant population differences. The nonmigratory *H. p. californicus* have high variation between populations, as evidenced by the disparate genetic diversities of SCA and WA populations (Table 2) and STRUCTURE. The migratory *H. p. purpureus* do exhibit site fidelity, which might explain the observed genetic differentiation. Studies on *H. p. purpureus* in Michigan (Magee, 1924) and New York (Yunick, 1987) show 56.8% and 23.6%, respectively (*n* = 1,770), of banded birds return to breed in the same area. Similar patterns of low population structure of highly migratory species are found in northern pike (*Esox lucius*) (Miller et al. 2001) and green turtles (*Chelonia mydas*) (Encalada et al., 1996).

Significant correlations were found between geographic and genetic distance in mtDNA and z‐linked loci, suggesting that physical distance could also restrict gene flow. Other barriers also play a role as some populations, WA and CoOR, that are geographically close are genetically differentiated. No obvious physical barriers exist between the WA and CoOR sites; however, contemporary gene flow could be restricted between these two populations due in part to plumage differences. Duvall (1945) noted a unique group of Pacific Northwest birds which he termed *Carpodacus purpureus rubidus* (not recognized as a subspecies by the American Ornithologists Union), in which males have darker heads, backs, rumps, and anterior lower parts; females are a darker olive. Both sexes lack mottling in the upper back and nape found in other nearby populations (Duvall, 1945). Similar morphological differences are present between the two recognized subspecies. *H. p. californicus* females are more olive green in plumage than *H. p. purpureus*, and *H. p. californicus* males have browner flanks and a duller red rump (Wootton, 1996). Isolation on small spatial scales has also been linked to the influence of suitable habitat on dispersal ability in boreal and black‐capped chickadees (*Poecile atricapillus*) (Adams & Burg, 2015; Adams, Lazerte, Otter, & Burg, 2016; Lait & Burg, 2013).

Behavior also plays a role in isolation, as behavioral differences exist with respect to song. The songs of the two subspecies are distinct, and the *H. p. purpureus* song is more variable (Wootton, 1996). As song is important in mate selection, it may reinforce reproductive isolation similar to assortative mating in *Ficedula* flycatchers (Qvarnström et al., 2010).

BARRIER did identify one barrier, which separates East Coast populations (NL and NS) from other populations. Although no obvious physical barrier is present, other studies found population structure in both animal and plant species in eastern Canada. Rueness et al. (2003) found reduced gene flow between eastern and western populations of Canada lynx (*Lynx canadensis*) south of James Bay, and in jack pine (*Pinus banksiana*) south of Hudson Bay (Jaramillo‐Correa, Beaulieu, Khasa, & Bousquet, 2009).

Our study has shown how the evolution of the purple finch has been influenced by a number of evolutionary processes and both historical and contemporary distributions. Prolonged isolation of the two subspecies was maintained during the last glacial maximum and contemporary physical barriers, and behavioral barriers including site fidelity, migration, and assortative mating, have maintained patterns of low gene flow between the two subspecies. Some secondary contact was found in a small portion of their range, although it was limited. The purple finch's evolutionary history demonstrates how population differences shape behavior and response to barriers to dispersal can influence evolution and, ultimately, speciation.

## Conflict of interest

We have no conflict of interest to declare.
